# Influence of Low Salt Concentration on Growth Behavior and General Biomass Composition in *Lyngbya purpurem* (*Cyanobacteria*)

**DOI:** 10.3390/md18120621

**Published:** 2020-12-04

**Authors:** Itzel Y. López-Pacheco, Susana Fuentes-Tristan, Laura Isabel Rodas-Zuluaga, Carlos Castillo-Zacarías, Itzel Pedro-Carrillo, María Adriana Martínez-Prado, Hafiz M. N. Iqbal, Roberto Parra-Saldívar

**Affiliations:** 1 Tecnologico de Monterrey, School of Engineering and Sciences, Monterrey 64849, Mexico; yolotzinlopez@tec.mx (I.Y.L.-P.); susy.fts@gmail.com (S.F.-T.); laura.rodas@tec.mx (L.I.R.-Z.); carloscastilloz@tec.mx (C.C.-Z.); 2 Chemical & Biochemical Engineering Department, Tecnológico Nacional de México-Instituto Tecnológico de Durango, Blvd. Felipe Pescador 1830 Ote. Durango, Durango 34080, Mexico; Itzelpedrocarrillo@gmail.com (I.P.-C.); adriana.martinezprado@itdurango.edu.mx (M.A.M.-P.)

**Keywords:** biomolecules, characterization, cyanobacteria, phycocyanin, salt influence

## Abstract

Cyanobacteria are essential for the vast number of compounds they produce and the possible applications in the pharmaceutical, cosmetical, and food industries. As *Lyngbya* species’ characterization is limited in the literature, we characterize this cyanobacterium’s growth and biomass. *L*. *purpureum* was grown and analyzed under different salinities, culture media, and incubation times to determine the best conditions that favor its cell growth and the general production of proteins, carbohydrates, lipids, and some pigments as phycocyanin and chlorophyll *a*. In this study, each analyzed biomolecule’s highest content was proteins 431.69 mg g^−1^, carbohydrates 301.45 mg g^−1^, lipids 131.5 mg g^−1^, chlorophyll *a* 4.09 mg g^−1^, and phycocyanin 40.4 mg g^−1^. These results can provide a general context of the possible uses that can be given to biomass and give an opening to investigate possible biocompounds or bio metabolites that can be obtained from it.

## 1. Introduction

Currently, cyanobacteria have risen in importance due to the many compounds produced by them and their possible applications in the food, energy, and pharmaceutical industries. The genus *Lyngbya* is an excellent example of cyanobacterial strains, showing extraordinary diversity in the production of more than 200 secondary metabolites with biological activity [1,2]. Some *Lyngbya* strains appear to be halophilic, adapting to different salt concentrations in the medium. For instance, the strain *Lyngbya contorta* shows better cell growth and protein production while increasing the salt concentration from 5 ppt to 40 ppt [3,4].

Several *Lyngbya* strains can produce pigments and bio-compounds with potential applications as pharmaceuticals due to their properties, including neuroprotective, anti-oxidant, antibacterial, antifungal, anti-inflammatory, antileishmanial, antidiabetic, and anticancer activity (Table 1). However, these possible applications in *L. purpureum* are still largely unknown [5,6,7,8]. Examples of these pigments are chlorophyll and phycocyanin, enabling cyanobacteria to efficiently use the light spectrum they are exposed to [9]. Single-cell protein (SCP) from *Lyngbya* and other cyanobacterial species are an alternative source of protein and other biomolecules. SCP can be used in human nutrition as protein supplements, animal breeding and cattle fattening, paper and leather processing, and other industries [10,11].

Other High-valuable products obtained from *Lyngbya* sp. are compounds with UV-protection properties [2,25,26]. Some of them are aromatic compounds like scytonemin and mycosporine-like amino acids [39,40,41]. Scytonemin has been found in *Lyngbya aestuarii*, [26,42], and *Lyngbya arboricola* [43].

Cyanobacterial strains are a potential source of carbohydrates. These carbohydrates consist mainly of glycogen and exopolysaccharide layers, which can be used for biofuels production [24]. Moreover, *Lyngbya* biomass has been used to obtain natural fibers with the same quality as the produced with jute and sisal fibers [44]. Regarding lipids, they are found widely in cyanobacteria’s membranes. For example, *L. limnetica* has lipidic productivity of 1.565 g L^−1^ d^−1^ [45].

*L. purpurem* is one of the *Lyngbya* species that has not been characterized yet in its general biomass composition and cell growth. This strain was first described in 1845 by Hooker and Harvey, who named it after the purple color they perceived in it, even though it was later discovered that *Lyngbya purpurem* acquires different pigmentations depending on the environmental conditions [46,47], being blue-green its most common color. This species’ lack of information and characterization allowed us to explore its general protein, carbohydrate, lipid, and pigments composition under low salt concentrations, different culture medium, and incubation time. All these experimentations to determine the best conditions to obtain higher biomass concentration and, subsequently, a vast amount of subproducts as expressed in Table 1 or otherwise not reported yet.

## 2. Results

### 2.1. Cell Growth of L. purpurem at Different Low Salt Concentrations and Culture Media

**Figure 1:**
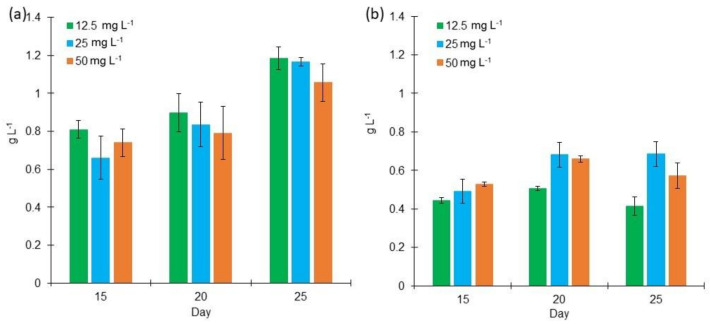
Cell growth of *Lyngbya purpurem* at different salt concentrations (Low: 12.5 mg L^−1^; Medium: 25 mg L^−1^; High: 50 mg L^−1^). (**a**) Dry weight of cultures growth in BG11 medium. (**b**) Dry weight of cultures growth in Bold 3N medium. All points were sampled by triplicate (*n* = 3).

By growing *L. purpurem* in different culture media, a clear difference in its growth is appreciated (Figure 1). In BG11 medium, the obtained biomass ranged from 0.65 g L^−1^ to 1.18 g L^−1^ during the days 15–25, while in Bold 3N medium, growth ranged between 0.41 g L^−1^ and 0.68 g L^−1^ on the same incubation time; being clear that *L. purpurem* grew better and faster in the BG11 medium. The BG11 medium has a higher nitrogen concentration compared to Bold 3N medium (1.5 g L^−1^ and 0.75 g L^−1^, respectively). Regarding BG11 medium (Figure 1a), the low and medium salt concentrations showed a better growth than that presented by the medium with high salt concentration, where the cyanobacterium starts showing an exponential behavior until day 25. The factorial design analysis shows that low and medium salt concentrations enhance cell growth at day 25. This growth observed in BG11 cultures was higher compared with previously reported in other studies [1]. On the other hand, *L. purpurem* produced 0.68 g L^−1^ of dry biomass in Bold 3N medium with a medium salt concentration on both 20th and 25th days (Figure 1b). Furthermore, it was determined that the best salt concentration is with medium salt concentration.

### 2.2. Proteins, Lipids, and Carbohydrates Content in L. purpurem’s Biomass at Different Low Salt Concentrations and Culture Media

**Figure 2:**
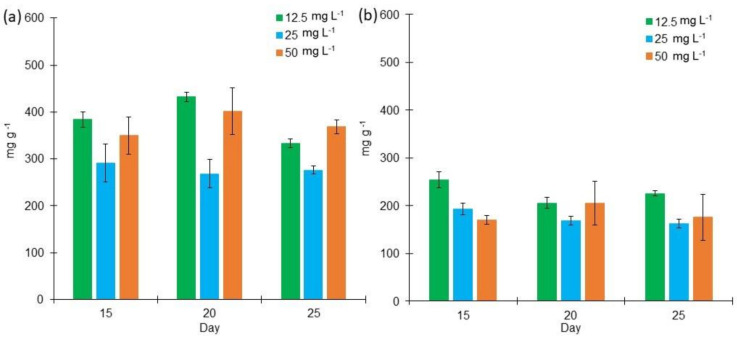
Protein content (mg g^−1^) of *Lyngbya purpurem* biomass at different salt concentrations (Low: 12.5 mg L^−1^; Medium: 25 mg L^−1^; High: 50 mg L^−1^) after 15, 20, and 25 days of growth. (**a**) Protein content of cultures growth in BG11 medium. (**b**) Protein content of cultures growth in Bold 3N medium. All points were sampled by triplicate (*n* = 3).

In order to analyze the possible applications of *L. purpureum* biomass, a general biomass characterization was performed. For this purpose, the protein content in the culture media was analyzed. BG11 cultures produced a higher protein content than the cultures grown in Bold 3N (Figure 2). In BG11 medium, the protein content ranged from 268.02 mg g^−1^ to 431.69 mg g^−1^ under different salinities between days 15 and 25. On the other hand, protein concentration in Bold 3N ranged from 162.22 mg g^−1^ to 253.71 mg g^−1^ during the same time length. The factorial design analysis illuminates that both, low and high salt concentrations, enhance protein content at day 20 in BG11 cultures. Also, it was discovered that low salinity enhances protein content at day 15 in Bold 3N cultures. It is interesting to observe that the highest protein content obtained from the biomass was 43.22% in BG11 medium and 27% in Bold 3N medium, which is higher than the observed in another study where *L. aestuarii* produced 13.5% DW [48]. Other cyanobacteria, such as *Arthrospira maxima*, have been reported to achieve a protein production of 50.7%. Also, *Leptolyngbya* sp. has produced 52.6% of proteins under salinities from 0 to 80 g/L [49].

**Figure 3:**
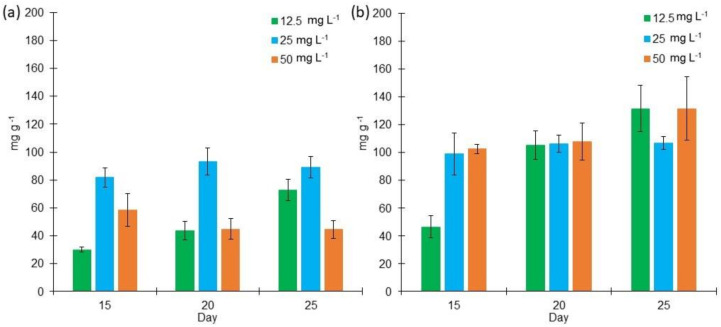
Lipid content (mg g^−1^) of *Lyngbya purpurem* biomass at different salt concentrations (Low: 12.5 mg L^−1^; Medium: 25 mg L^−1^; High: 50 mg L^−1^) after 15, 20, and 25 days of growth. (**a**) Lipid content of cultures grown in BG11 medium. (**b**) Lipid content of cultures grown in Bold 3N medium. All points were sampled by triplicate (*n* = 3).

Bold 3N cultures produced a higher lipid content than the cultures grown in BG11 (Figure 3). In BG11 medium, the lipid content varied from 29.84 mg g^−1^ to 93.12 mg g^−1^ under different salinities at days 15–25, while in Bold 3N medium, lipid content ranged from 46.45 mg g^−1^ to 131.55 mg g^−1^ during the same period.

The factorial design analysis of the BG11 cultures determined that medium salt concentration enhances lipid content at day 20. This lipid content corresponded to 9.23% of the total biomass weight. In the case of Bold 3N cultures, it was determined that high salinity enhances lipid content at day 25. At this point, lipid concentration was 12% of the total DW of the biomass.

**Figure 4:**
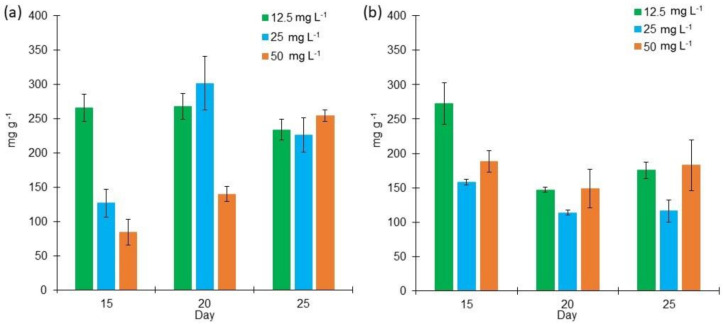
Carbohydrate content (mg g^−1^) of *Lyngbya purpurem* biomass at different salt concentrations (Low: 12.5 mg L^−1^; Medium: 25 mg L^−1^; High: 50 mg L^−1^) after 15, 20, and 25 days of growth. (**a**) Carbohydrate content of cultures grown in BG11 medium. (**b**) Carbohydrates content of cultures grown in Bold medium. All points were sampled by triplicate (*n* = 3).

The total carbohydrate content was analyzed. The highest concentrations were found in the cultures grown at a high salt concentration in Bold 3N culture (271.83 mg g^−1^) and in BG11 in medium salt concentration (301.45 mg g^−1^) (Figure 4). The average carbohydrate production from days 15 to 25 in BG11 was 210.94 mg g^−1^, while Bold 3N, carbohydrate production was 166.74 mg g^−1^ (Figure 4a,b). From the factorial design analysis of the BG11 cultures, medium salt concentration enhances carbohydrate content at day 20 (29% of the total DW of biomass). Regarding the Bold 3N cultures, low salt concentration enhances carbohydrates production at day 15 (27% of the total DW of biomass).

### 2.3. Pigments Behavior in L. purpurem’s Biomass Different Low Salt Concentrations and Culture Media

**Figure 5:**
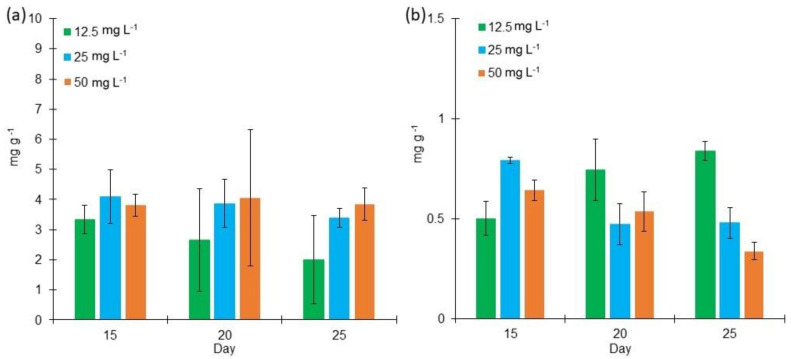
Chlorophyll content (mg g^−1^) of *Lyngbya purpurem* biomass at different salt concentrations (Low: 12.5 mg L^−1^; Medium: 25 mg L^−1^; High: 50 mg L^−1^) after 15, 20, and 25 days of growth. (**a**) Chlorophyll *a* content of cultures growth in BG11 medium. (**b**) Chlorophyll *a* content of cultures growth in Bold 3N medium. All points were sampled by triplicate (*n* = 3).

One of the most common pigments produced by *L. purpurem* is chlorophyll *a* (Figure 5). The highest contents were found in BG11 cultures in contrast to 3N Bold cultures. The highest concentrations were found in the cultures grown in the medium salt concentration on both culture media: 4.09 mg g^−1^ produced in BG11 on day 15, and 0.38 mg g^−1^ produced in Bold 3N on day 15. From the factorial design analysis of BG11 cultures, it was determined that neither of the two factors (salt concentration nor incubation time length) is essential for chlorophyll *a* production. For Bold 3N cultures, it was seen that low and medium salt concentrations enhance chlorophyll *a* content at day 25 and 15, respectively.

**Figure 6:**
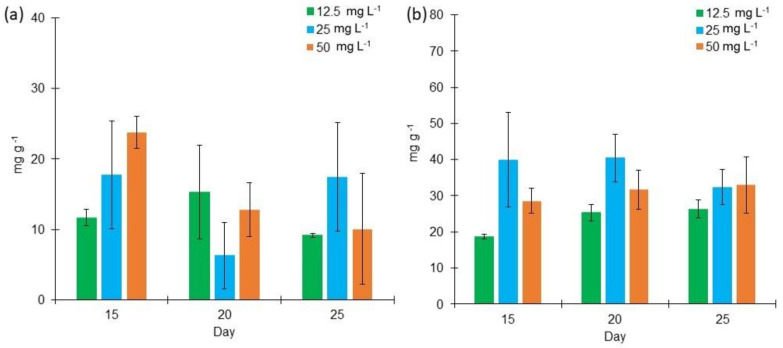
Phycocyanin content (mg g^−1^) of *Lyngbya purpurem* biomass at different salt concentrations (Low: 12.5 mg L^−1^; Medium: 25 mg L^−1^; High: 50 mg L^−1^) after 15, 20, and 25 days of growth. (**a**) Phycocyanin content of cultures grown in BG11 medium. (**b**) Phycocyanin content of cultures growth in Bold 3N medium. All points were sampled by triplicate (*n* = 3).

Even though previous studies show that *Lyngbya* sp. produces phycobiliproteins as the phycocyanin [25,50], neither the amount of phycocyanin produced nor the factors that enhance its production have been reported. From the two different culture media used, Bold 3N enhances a higher phycocyanin production ranging from 18.6 mg g^−1^ to 40.4 mg g^−1^, whereas with BG11, phycocyanin ranged from 6.2 mg g^−1^ to 23.7 mg g^−1^ (Figure 6). Our results can be compared to other studies where 20.43 g of phycocyanin was produced by *Lyngbya* sp. A09DM at incubation day 30 [19]. *N. muscorum* and *P. foveolarum* were also exposed to different salt concentrations, producing phycocyanin in a range of concentration from 30 mg g^−1^ to 70 mg g^−1^, approximately [51].

## 3. Discussion

### 3.1. Cell Growth of L. purpurem at Different Low Salt Concentrations and Culture Media

BG11 medium contains an inorganic carbon source (Na_2_CO_3_ 20 mg L^−1^). This amount of nitrogen and the inorganic carbon source can positively affect the growth of the microalgae. Therefore, better growth of *L. purpurem* was obtained using the BG11 culture medium. The total dry biomass presented in this work resulted higher than the 0.642 g L^−1^ of *Lyngbya*’s biomass previously reported in the literature, where the BG11 medium did not contain any variation in the salt concentration [52]. Moreover, some studies have reported the growth of *L. aestuarii* in f/2 medium with a salinity of 0.03 ppb, producing 0.0024 g L^−1^ at day 30 (Shruthi & Rajashekhar, 2014). The biomass growth results in BG11 and Bold 3N at different salt concentrations give the possibility of using these media to obtain an optimal growth of *L. purpurem* and marine *Lyngbya* species. Compared to other cyanobacteria, the obtained biomass of *L. purpureum* can be compared with the 1.45 g L^−1^ and 1.63 g L^−1^ obtained by *Spirulina subsalsa* BDU51591 and *Oscillatoria* sp. BDU51731, respectively [53]. Although the salt concentrations used in this study were deficient and not comparable to seawater, this cyanobacterium has the potential to be used at higher salinities, which can be explored in further studies.

Furthermore, cyanobacteria production in seawater can be an excellent option to produce biofuels and metabolites of interest [54]. Also, to counteract the salty environment’s stress, cyanobacteria prevent the entry of Na^+^ ions into the cell using Na^+^/H^+^ antiporters and K^+^ transporters or by accumulating solutes as sucrose, trehalose, glucosyl glycerol, proline, and glycine betaine. Additionally, exopolysaccharides (EPSs) protect cyanobacteria against osmotic and salt stress [55]. Therefore, the NaCl concentrations could influence the biomass content.

### 3.2. Protein, Lipid, and Carbohydrate Contents in L. purpurem’s Biomass at Low Salt Concentrations and Culture Media

It can be observed that independently of the salt concentration evaluated; there was a higher protein production in the BG11 culture medium than in Bold 3N medium. The high amount of protein produced may result from the nitrogen concentration used in BG11, which was twice the concentration used in the Bold 3N medium (1.5 g L^−1^ and 0.75 g L^−1^, respectively). It has been reported before that a higher level of nitrogen stimulates the production of proteins since nitrogen is one of the principal components in amino acids [56,57]. Their proteins also tend to have high lysine content, making them suitable for food supplements [58].

Regarding the total production of lipids, the highest concentration was obtained using the Bold 3N medium at the low salt concentration on days 20 and 25. Similar to our results, other studies have evaluated the response to nitrogen starvation in microalgae like *Chlorella* sp. [59], *Chlorella vulgaris* [60], *Scenedesmus* sp. [61], and *P. tricornutum* [62], finding a lipid accumulation. For instance, Chandra et al. reported a higher lipid and FAMES production performed by *Lyngbya* sp. using a culture media with low nitrogen concentration [63]. Also, our results show a more significant lipid production (131.5 mg g^−1^) compared to the produced by *Chlorella* sp. (85mg g^−1^) and *Ankistrodesmus* sp. (81 mg g^−1^) grown in the BG11 culture medium.

It is important to highlight that our lipids production was higher than most previous studies in the literature. For example, *Lyngbya* sp. showed a lipid production of 102.95 mg g^−1^ at day 19 [52], and *L. dendrobia* produced a total lipid content of 10.55% when it is cultured in BG11 medium. Similarly, *L. limnetica*, cultured in BG11, produced a lipidic content of 18.1% [64], and *L. aestuarii* produced 14.4% DW of lipids when it was grown in f/2 medium [48]. Among the lipids that have been found in *Lyngbya*’s biomass, saturated and unsaturated FAME can be used as biofuels or for animal feed. For example, Chandra et al. reported palmitic and linoleic acid production. On the one hand, palmitic acid has antimicrobial properties; on the other hand, linolenic acid has been used as a dietary supplement. [63,65]. Compared to other cyanobacteria, the lipids produced in our study are comparable to the obtained by *Chroococcus* sp. BDU20201 (5.79%), *Spirulina subsalsa BDU51591* (18.51%), *Oscillatoria salina* BDU50701 (11.9%), and *Oscillatoria* sp. BDU51731 (16%), making this cyanobacterium a good option for obtaining biofuels. Our findings are also consistent with the results obtained with other *Lyngbya* strains, where 4–12% DW belongs to lipids [53].

Our results show a higher carbohydrate production in comparison to other strains of *Lyngbya* sp. For instance, the total carbohydrate content of 172.89 mg g^−1^ in *Lyngbya* sp. [52] was obtained, which is similar to the 182 mg g^−1^ DW obtained in our study at day 25 in Bold 3N added with a high salt concentration. Also, *L. aestuarii* obtained an 18.4% of carbohydrates when it was grown in f/2 medium [48]. This result is similar to the obtained by *L. purpurem* in our study with BG11 medium (29% of the total DW of biomass). Large amounts of sucrose have been found in cyanobacteria; this carbohydrate is the most used for biofuels production. It was observed that the addition of optimal amounts of NaCl to enhance sucrose accumulation by *Synechocystis* sp. PCC6803. [66]. The total carbohydrate content from microalgae biomass varies between 3% and 40% [58], which supports our results (27% and 29%), which means that *Lyngbya purpurem* can produce one of the highest carbohydrate contents among different microorganisms.

### 3.3. Pigments Behavior in L. purpurem’s Biomass Different Low Salt Concentrations and Culture Media

The chlorophyll-*a* production has been reported in other cyanobacteria such as *Spirulina platensis*, finding a production of 4.28 mg g^−1^ [67], 11.5 mg g^−1^ [68], and 12.3 mg g^−1^ [69]. These represent a higher production than those obtained in our study. The cyanobacteria *N. muscorum* and *P. foveolarum* have been exposed to different salt concentrations where chlorophyll *a* achieved a production range from 14 mg g^−1^ to 20 mg g^−1^, approximately [51]. Furthermore, it has been shown that salt concentration can affect the chlorophyll-*a* structure negatively in different cyanobacteria such as *Spirulina platensis* [70].

It is interesting to observe that although a higher biomass growth and chlorophyll content were obtained in BG11 medium, in terms of phycobiliprotein production, the best culture medium was Bold 3N. From the factorial design analysis of Bold 3N cultures, it was determined that salt concentration is essential in phycocyanin production. Setyoningrum & Nur reported that an increase in salt concentration causes a significant increment in *Spirulina maxima*’s phycocyanin production to answer stressful environmental conditions [71]. Table 2 summarizes all the optimal growth conditions found in the present study.

## 4. Materials and Methods

### 4.1. Reagents and Equipment

The reagents used for the culture medium, and the lipids and carbohydrates analysis were purchased from Sigma-Aldrich (Saint Louis, MO, USA). The Lowry Protein Assay Kit was purchased from Thermo Fisher Scientific (Rockford, lL, USA). The spectrophotometric measurements were taken in an absorbance microplate reader BGM Labtech (Fluorstar omega, 415-0470, Ortenberg, Germany).

### 4.2. Culture Conditions

**Figure 7:**
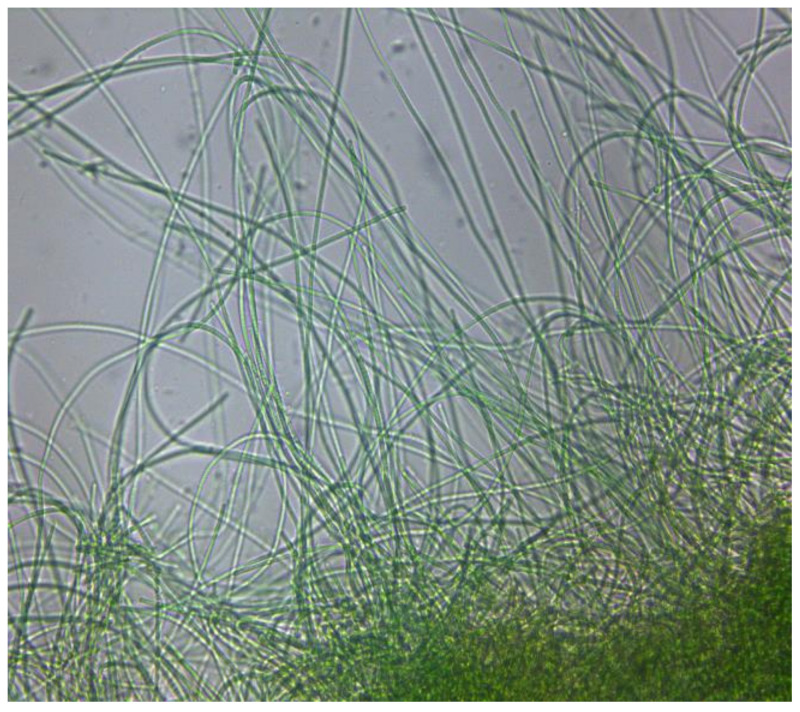
*Lyngbya purpurem* LB 2716 in BG11 Medium (40X).

The strain *Lyngbya purpurem* LB 2716 was purchased from UTEX (UTEX, Austin, TX, USA) (Figure 7). Erlenmeyer flasks were adapted in a shaker at 160 rpm (Scientific industries, SI1600 Orbital-genie, NY, USA) with a photoperiod of 12:12 using LED lamps (Hydro Grow LED, Los Angeles, CA, USA) with a light intensity of 100 μmol m^−2^ s^−1^. The temperature of the incubation room was 21 °C. Two different culture media were used: modified BG11 and Bold 3N. The BG11 culture medium contained: NaNO_3_ 1.5 g L^−1^, K_2_HPO_4_ 40 mg L^−1^, CaCl_2_·2 H_2_O 36 mg L^−1^, MgSO_4_·7 H_2_O 75 mg L^−1^, Citric Acid·H_2_O 6 mg L^−1^, Ferric Ammonium Citrate 6 mg L^−1^, Na_2_EDTA·2 H_2_O 1 mg L^−1^, Na_2_CO_3_ 20 mg L^−1^, H_3_BO_3_ 2.86 mg L^−1^, MnCl_2_·4 H_2_O 1.81 mg L^−1^, ZnSO_4_·7 H_2_O 0.22 mg L^−1^, Na_2_MoO_4_·2 H_2_O 0.39 mg L^−1^, CuSO_4_·5 H_2_O 0.079 mg L^−1^ and Co(NO_3_)_2_·6 H_2_O 0.0494 mg L^−1^. The Bold 3N culture medium contained: NaNO_3_ 0.75 g L^−1^, K_2_HPO_4_ 75 mg L^−1^, CaCl_2_·2 H_2_O 25 mg L^−1^, MgSO_4_·7 H_2_O 75 mg L^−1^, KH_2_PO_4_ 0.175 g L^−1^, Na_2_EDTA·2 H_2_O 4.5 mg L^−1^, FeCl_3_·6 H_2_O 0.582 mg L^−1^, MnCl_2_·4 H_2_O 0.246 mg L^−1^, ZnCl_2_ 0.03 mg L^−1^, CoCl_2_·6 H_2_O 0.012 mg L^−1^, Na_2_MoO_4_·2 H_2_O 0.024 mg L^−1^.

### 4.3. Cell Growth of L. purpurem at Different Salt Concentrations and Culture Medium

For each experiment, 0.30 g DW of wet biomass were used as seed culture and were inoculated into a 250 mL Erlenmeyer flask with the corresponding culture medium, resulting in 150 mL of total volume. A Factorial Design was used for this research and was applied to each set of experiment (Table 3). Cell growth and biomass composition of *L. purpurem* was evaluated in 2 different culture media (BG11 and Bold 3N) under 3 different salt concentrations: low (12.5 mg L^−1^), medium (25 mg L^−1^), high (50 mg L^−1^), during 3 different incubation time lengths (15, 20, and 25 days).

The culture medium Bold 3N is often used to grow *L. purpurem.* Its original composition contains 25 mg L^−1^ of NaCl, so this value was taken as a reference to set the different salinities used in this study. At incubation days 15, 20, and 25, the biomass of all salt concentrations (low, medium, and high) was dried at 4 °C for two days. The dry biomass (DB) was weighted and resuspended in 20 mL of bi-distilled water. The weight of DB was measured to obtain the cell growth behavior.

### 4.4. Proteins, Lipids, and Carbohydrates Content in L. purpurem’s Biomass at Different Salt Concentrations and Culture Medium

The DB was resuspended in 20 mL of bidistilled water and was used to quantify the proteins, lipids, carbohydrates and pigments present in the biomass. Each sample (20 mL) was sonicated in a Sonifier Cell Disruptor (Branson Model # 102 C) to achieve cellular lysis. After this, the biomass protein content was quantified using the Lowry Protein Assay Kit (Lot # RB230458A), the lipid content was quantified using the sulfo-phospho-vanillin assay described by Mishra et al. [72] and the carbohydrate content was quantified using the sulfuric acid-UV assay described by López-Legarda et al. [73].

### 4.5. Pigments Behavior in L. purpurem Biomass at Different Salt Concentrations and Culture Medium

Each sonicated sample was also used to determine the pigments concentration. 5 mL of each sample were dried at 40 °C for two days, followed by a resuspension in 5 mL of methanol (95%) and incubation at 60 °C for 5 min. The samples were centrifuged for 10 min at 4000 rpm and 15 °C. The chlorophyll *a* concentration was obtained by measuring the supernatant’s absorbance at 652 and 665 nm wavelengths by spectrophotometry. The concentration was calculated with the following formula described by [74,75]:

(1) $$\mathrm{Chl}\;a=16.29\;A^{665}-8.54\;A^{652}$$

To evaluate the phycocyanin concentration, the DB was resuspended in 20 mL of bidistilled water after sonication. The phycocyanin concentration was obtained by measuring the supernatant’s absorbance at 592, 618, and 645 nm wavelengths by spectrophotometry. The concentration was calculated with the following formula described by [76]:

(2) $$\mathrm{PC}\;\left({\mathrm{mg}\;\mathrm{mL}}^{-1}\right)=[(A^{618}-A^{645})-\left(A^{592}-A^{645}\right)0.51]0.15$$

### 4.6. Data Analysis

All data were analyzed in Minitab 18 (Minitab Inc., State College, PA, USA). A Multi-Factor ANOVA with a Tukey pairwise comparison per type of culture media was used in order to analyze the factorial design data. The coated values were taken from the triplicate samples (*n* = 3) and expressed as +/− SD in Figures and Tables.

## 5. Conclusions

This study reports the first characterization of the biomass of *Lyngbya purpurem* under different culture conditions. This study was conducted in order to elucidate the optimal conditions to produce several high-value biomolecules. Total biomass production of 1.18 g L^−1^ was achieved at low salinity in BG11 medium on the 25th incubation day. The highest production of each analyzed biomolecule in this study was: protein 431.69 mg g^−1^ (BG11, low salinity at the 20th day), carbohydrate 301.45 mg g^−1^ (BG11, medium salinity at the 20th incubation day), lipid 131.5 mg g^−1^ (Bold 3N, low salinity at the 25th incubation day), chlorophyll *a* 4.09 mg g^−1^ (BG11, medium salinity at the 15th incubation day), and phycocyanin 40.4 mg g^−1^ (Bold 3N, medium salinity at the 20th day).

*Lyngbya purpurem* has shown to be an adequate protein and carbohydrates source, which can be used in the food industry as a food supplement for humans or animal feed. *L. purpurem*’s biomass has high nutritional value due to its protein, carbohydrate, and lipid content. Furthermore, it is important to highlight the antioxidant and anticancer properties conferred by chlorophyll *a*, namely the anti-inflamatory, anticancerous, hepatoprotective, and neuroprotective activities of phycocyanin. As a natural bio-factory, *L. purpurem*’s carbohydrates and lipids can also be used for biofuels and bioplastic production. Other applications, such as biofertilizer, nutraceutical, and biomaterials, can be evaluated as part of a future study to impact the pharmaceutical, nutraceutical, agricultural, and cosmetic industries.

## Figures and Tables

**Table 1:** Beneficial properties and applications of *Lyngbya* biomolecules.

Biomolecule	Beneficial Properties and Applications	Reference
General biomolecules of *Lyngbya* species		
Chlorophyll *a*	Potential applications in the cosmetic, food, and pharmaceutical industries due to its anticarcinogenic and antimutagenic activity, and inhibitory effect on genotoxicity. It has been shown to reduce acne lesions and sebum levels, exhibiting antioxidant activity and protection against lipid oxidation at concentrations of 1mM L^−1^.	[12,13,14,15]
Phycocyanin	Applications as fluorescent agent in fluorescent probes; in food preservation by working as antioxidant; as natural pigment in food, beverages, cosmetics, and textiles; and as nutraceutical and pharmaceutical due to its anti-inflamatory, antioxidant, anticancerous, hepatoprotective, neuroprotective, and hypocholesterolemic activity.	[16,17,18,19,20,21]
Protein	Protein production from microbial biomass, or single-cell protein (SCP), as an alternative source of protein and other biomolecules rather than animal sources. SCP can be used in human nutrition as protein supplements, in animal breading and cattle fattening. Furthermore, in paper, leather processing, and other industries.	[10]
Lipids	Lipids produced by microorganisms can be used in the production of biodiesel and other biofuels.	[22,23]
Carbohydrates	Microbial carbohydrates can be used for biofuels production and food industry as an alternative source of carbohydrates production.	[24]
Specific biomolecules of *Lyngbya* species		
Scytonemin and Mycosporine-like aminoacids	UV-absorbing compounds with potential applications in cosmeceuticals as sun-care and antiaging products, and photostabilizer additives in paints, varnishes, and plastics. They possess antioxidant activity and antiphotoaging effect on human skin cells.	[2,25,26]
Malyngolide	Antibacterial and antifungal activity	[27]
2,5-dimethyldodecanoic acid	Herbicidal activity	[28]
Madangolide	Cytotoxicity against several cancer cell lines	[29]
Laingolide A	Cytotoxicity against several cancer cell lines	[29]
Lobocyclamide	Antifungal activity	[30]
Pompanopeptin A (PPPA)	Inhibitor of trypsin	[31]
Sulfolipids with different fatty acid esters	Anti-HIV activity	[32]
Debromoaplysiatoxin	Dermonecrotic activity and inflammatory	[33]
Carmabin A	Antimalarial activity	[34,35]
Curacin A	Cytotoxicity against several cancer cell lines	[34]
Tanikolide	Cytotoxicity against several cancer cell lines	[36]
Antillatoxin	Sodium-channel-blocking activity	[37]
Apratoxin D	Cytotoxicity against several cancer cell lines	[38]

**Table 2:** Optimal conditions for the highest production yield of proteins, lipids, carbohydrates, chlorophyll *a* and *b*, phycocyanin, and biomass by *Lyngbya purpurem*.

	BG11		Bold 3N		Production Yield	
	Salinity	Incubation Time	Salinity	Incubation Time		
Total biomass	Low	25			1.18 ± 0.06	g L^−1^
Proteins	Low	20			431.69 ± 9.5	mg g^−1^ DW
Lipids			Low	25	131.5 ± 22.81	mg g^−1^ DW
Carbohydrates	Med	20			301.45 ± 38.94	mg g^−1^ DW
Chlorophyll *a*	Med	15			4.09 ± 0.88	mg g^−1^ DW
Phycocyanin			Med	20	40.4 ± 2.23	mg g^−1^ DW

Salinity: Low: 12.5 mg L^−1^; Medium: 25 mg L^−1^; High: 50 mg L^−1^. Incubation time: cultivation days. (*n* = 3).

**Table 3:** Experimental design for the salt concentration influence on growth behavior and biomass composition in *L. purpurem*.

Run	Blocks	Salinity	Day
1	1	Low	15
2	1	Low	20
3	1	Low	25
4	1	Medium	15
5	1	Medium	20
6	1	Medium	25
7	1	High	15
8	1	High	20
9	1	High	25
10	2	Low	15
11	2	Low	20
12	2	Low	25
13	2	Medium	15
14	2	Medium	20
15	2	Medium	25
16	2	High	15
17	2	High	20
18	2	High	25
19	3	Low	15
20	3	Low	20
21	3	Low	25
22	3	Medium	15
23	3	Medium	20
24	3	Medium	25
25	3	High	15
26	3	High	20
27	3	High	25

Salinity: Low: 12.5 mg L^−1^, Medium: 25 mg L^−1^, High: 50 mg L^−1^. Factorial Design was run per type of culture medium (BG11 and Bold 3N).
